# Mindfulness and media-driven prosociality: effects of trait and state mindfulness on responses to conflict photojournalism

**DOI:** 10.3389/fpsyg.2025.1619688

**Published:** 2025-08-13

**Authors:** Siqi Cheng, Sixiang Liu, Xuan Zhang, Jing Zhou, Xinran Feng

**Affiliations:** ^1^College of Journalism and Communication, Jinan University, Guangzhou, China; ^2^International School, Jinan University, Guangzhou, China; ^3^Department of Psychological and Cognitive Sciences, Tsinghua University, Beijing, China

**Keywords:** trait mindfulness, state mindfulness, media-driven prosociality, war and conflict photojournalism, psychological distance

## Abstract

In a year where war and conflict are primarily mediated through emotionally powerful images, maintaining audience engagement without inducing compassion fatigue remains a key challenge. This study examines how trait mindfulness (a stable characteristic) and state mindfulness (induced through short- or long-term meditation) affect psychological closeness, empathy, and prosocial behavior in response to conflict photojournalism. Across three studies, we find that individuals with higher levels of trait mindfulness show greater empathy and willingness to help, even without prior meditation practice. Short-term mindfulness interventions reduce psychological distance, while long-term training enhances costly prosocial behaviors (e.g., donations, volunteering), though empathy is not necessarily increased. These results suggest that mindfulness, whether innate or trained, can enhance media-driven prosocial responses, with effects varying based on individual traits and intervention duration. The findings contribute to media psychology by revealing how mindfulness shapes the emotional and behavioral impact of war journalism, offering practical implications for ethical media presentation and audience engagement.

## 1 Introduction

It has been argued that a war without images is virtually unimaginable in today’s world (Müller, 2007), highlighting the increasingly visual nature of war and conflict coverage. For most audiences, wars and crises are experienced as distant events—encountered solely through media—and news images are often perceived as objective reflections of reality (Huxford, 2001). Due to their inherent capacity to convey powerful emotions, photojournalistic images not only attract attention and raise awareness of war, conflict, and disaster, but also enable emotional engagement with distant suffering by capturing the intensity and drama that words alone often fail to express (Dobernig and Lobinger, 2010). In particular, images that depict destruction and humanitarian suffering are thought to more effectively elicit empathy for distant victims (Weikmann and Powell, 2019), potentially fostering prosocial behaviors such as charitable giving, peace advocacy, and political participation (Kaplan, 2011).

However, some researchers caution that the constant exposure to images of suffering may result in compassion fatigue, a state of emotional numbing or desensitization in which audiences become less responsive to others’ pain (Tester, 2001). Others argue that people may still care but feel helpless to intervene, leading them to disengage emotionally or avoid exposure to distressing content (Kaplan, 2008). From this perspective, compassion fatigue is not merely a lack of empathy, but a self-protective mechanism that suppresses moral impulses in order to avoid emotional overload (Cameron and Payne, 2011). This raises an important concern: how can we help media audiences remain emotionally connected and willing to act, rather than retreat from the suffering they witness?

One promising approach lies in mindfulness, which may enhance individuals’ emotional regulation and reduce the psychological defenses that lead to disengagement. Originally rooted in religious traditions, mindfulness meditation has since been adopted as a secular strategy for psychological well-being (Dunlop, 2015). Research has shown that mindfulness-based practices are associated with greater empathy and reduced compassion fatigue, with benefits observed in caregivers, trauma responders, and health professionals (Najjar et al., 2009; Fortney et al., 2013; Liu et al., 2025a,b). This suggests that mindfulness might also help media audiences maintain emotional engagement and prosocial concern in response to distant suffering portrayed in war photojournalism.

Mindfulness is generally understood in two forms: trait mindfulness, a stable individual disposition, and state mindfulness, a temporary condition cultivated through practice (Friese and Hofmann, 2016). Both have been linked to increased empathy and prosocial behavior (Winning and Boag, 2015; Donald et al., 2019). However, their distinct roles in shaping responses to media content, especially under morally and emotionally demanding conditions, remain underexplored.

Against this background, the present research investigates whether mindfulness (both as a trait and a trainable state) can promote psychological closeness, empathy, and helping behaviors in the context of war photojournalism. We address three research questions: First, without mindfulness training, are individuals with higher trait mindfulness more likely to experience greater psychological closeness, empathy, and prosocial behavior toward victims portrayed in war photojournalism? Second, from an intervention perspective, does short-term state mindfulness training enhance these outcomes, thereby influencing media effects? Third, does the duration of mindfulness practice play a role, specifically, does longer-term meditation training result in greater emotional engagement and prosocial responses? Through three empirical investigations, this study explores how mindfulness shapes media audiences’ emotional and behavioral responses to images of war and conflict. The findings offer practical implications for journalism, suggesting that integrating mindfulness-informed strategies into news production and audience engagement may help enhance empathetic resonance, and promote more sustained public concern for distant suffering.

## 2 Literature review

### 2.1 War and conflict photojournalism and prosociality

War and conflict journalism is a branch of journalism that conveys news about war and conflict through images. Research in this field primarily centers on two areas: one focuses on the ethical principles guiding professional photojournalists, and the other examines how audiences interpret meaning through different visual frames and the media effects they produce.

In studies concerning professional ethics, a key perspective holds that images not only enhance our understanding of distant others, but also shape our emotional responses and attitudes toward them (Silverstone, 2013). This capacity places an ethical obligation on photojournalists, requiring them to uphold responsibilities such as truthfully representing reality, respecting and protecting those who suffer, and avoiding unnecessary intrusion into their privacy (Stupart, 2021; Maciá-Barber, 2013). For instance, some scholars have questioned whether the use of filters in war photography on social media risks aestheticizing suffering, thus potentially undermining the credibility and professionalism of photojournalism (Alper, 2014).

Research on visual framing begins with comparisons between the effects of photojournalism and written journalism. Empirical findings suggest that, in the context of war coverage, images tend to be more impactful than text. Photographs depicting war casualties are more likely to provoke public opposition to war, as well as feelings of confusion and sorrow, compared to textual reports alone (Pfau et al., 2006). Additionally, studies show that multimodal presentations (e.g., combining text and images) produce more complex effects: while textual framing influences support for policy, images evoke emotional reactions such as fear and anger, which can in turn affect behavioral intentions (Powell et al., 2015). Another important strand of research examines the effects of different types of visual frames. For example, Midberry (2020) categorized U.S. war images into two primary visual frames: militarism and the human cost of war. The former typically features U.S. soldiers and military equipment to highlight strength and power, while the latter includes visuals such as reactions to loss, civilian casualties, and military deaths. Further research by Gartner (2011) found that audiences shown images of human cost were more likely to express anti-war sentiments than those exposed to militarism images.

Taken together, the overarching goal of research on war photojournalism is to awaken empathy for distant suffering and promote anti-war attitudes. Numerous studies have confirmed a direct connection between the media’s portrayal of suffering and the audience’s prosocial responses, such as empathy and moral concern (Wald et al., 2021; Dumitrescu and Bucy, 2021; Iyer et al., 2014). However, recent findings suggest that the compassion and concern evoked through repeated exposure to human suffering in the news may be short-lived. Over time, audiences tend to experience compassion fatigue, becoming emotionally desensitized in the face of increasingly frequent depictions of suffering (Tester, 2001; Kaplan, 2008).

Therefore, in evaluating the potential of war and conflict photojournalism to foster prosocial behavior, it is crucial not only to adhere to ethical principles and improve the presentation of visual frames, but also to address the decline in empathy caused by compassion fatigue. Some studies have found that, rather than focusing solely on scenes of suffering, portraying victims in relation to their everyday lives, such as their families or careers, can more effectively counteract compassion fatigue and foster deeper social connection (Roberts, 2021). This suggests that compassion fatigue can be mitigated, and that with thoughtful framing, war photojournalism can continue to serve as a powerful tool for promoting empathy and social awareness.

### 2.2 State mindfulness, trait mindfulness and prosociality

Mindfulness meditation originated from Buddhist and Hindu contemplative practices and is commonly defined as a non-judgmental awareness and focused attention on present-moment cognitive, emotional, and sensory experiences, without attachment to thoughts about the past or future (Kabat-Zinn, 1990). As techniques have evolved, mindfulness has increasingly been conceptualized as a meditative practice integrating attention regulation, emotional control, and self-observation. It is now recognized as an effective intervention in both psychological therapy and the treatment of physical illnesses (Lutz et al., 2016). The most widely adopted mindfulness-based program is Jon Kabat-Zinn’s Mindfulness-Based Stress Reduction (MBSR), which includes breathing exercises, body scanning, and yoga (Kabat-Zinn, 1990), as well as its derivative, Mindfulness-Based Cognitive Therapy (MBCT) (Teasdale et al., 1995). The mindfulness state achieved through such meditative practices is referred to as state mindfulness (Bohlmeijer et al., 2010). The previous studies have demonstrated that state mindfulness training in daily life can effectively reduce stress and fatigue (Janssen et al., 2018), alleviate anxiety and depression (Arch and Craske, 2010; Liu et al., 2025a,b), and improve conditions such as sleep disorders (Larouche and Araújo-Oliveira, 2014) and anorexia nervosa (Cowdrey and Park, 2012). Particularly during the COVID-19 pandemic, mindfulness played a critical role in mitigating psychological distress caused by the crisis (Belen, 2022).

In addition to the self-oriented benefits of state mindfulness meditation, an increasing number of studies have begun to explore its other-oriented effects, particularly its role in fostering prosocial behaviors such as empathy, caring for others, and offering help. Empathy, the capacity to understand others’ emotional states and motivations, adopt their perspectives, and accept their viewpoints, is a crucial component of social cognition. It enables individuals to respond effectively to others’ emotions and engage in emotional communication (De Waal, 2008). Research has shown that mindfulness can enhance individuals’ empathic capacity, increasing attention to and understanding of others (Trent et al., 2016). It can also foster empathetic concern for others’ suffering and motivate actions to alleviate their distress (Himichi et al., 2021). Several studies have explored the mechanisms by which mindfulness may promote voluntary altruistic behavior. One line of research suggests that mindfulness shifts attention from self-centered needs to the needs of others (Van Doesum et al., 2020). Another view posits that mindfulness enhances overall well-being, which in turn provides intrinsic motivation for prosocial behavior (Luberto et al., 2018).

Furthermore, while long-term meditation training has been shown to improve mental health, strengthen social identity, and encourage altruistic actions, short-term interventions also demonstrate positive effects on psychological adjustment and prosocial tendencies (Malin and Gumpel, 2022; Liu et al., 2020). For example, Hafenbrack et al. (2020) found that just five consecutive days of a single 7–15 min session of positive breathing practice per day significantly promoted prosocial behaviors, such as increased financial generosity. Even less than an hour of intermittent mindfulness intervention has been shown to produce significant positive effects on prosociality (Donald et al., 2019).

Mindfulness, in a broader sense, encompasses not only the state cultivated through meditation but also a trait, a dispositional tendency to remain mindful in everyday life, which is relatively stable within an individual’s personality (Baer et al., 2006). Trait mindfulness has been positively associated with self-awareness, self-regulatory behavior, psychological resilience, positive affect, subjective well-being, and satisfaction in work and life domains (Bergomi et al., 2013; Short et al., 2016). Individuals high in trait mindfulness are not only more attuned to their own emotional states but also exhibit greater sensitivity to and attention toward emotional cues in others (Brown and Ryan, 2003). Moreover, a growing body of research supports a positive association between trait mindfulness, empathy, and prosocial behavior (Borghi et al., 2023). However, some researchers have found no significant link between trait mindfulness and specific forms of prosocial behavior, such as charitable donations or altruistic behavior in incentivized economic games. These findings suggest that the role of trait mindfulness in promoting prosocial behavior may be more nuanced and context-dependent (Schindler and Pfattheicher, 2023).

Some scholars argue that both state mindfulness and trait mindfulness can be cultivated through meditation practice. On one hand, a single session of meditation can temporarily alter brain states and modulate related neural activity or connectivity patterns. On the other hand, repeated meditation practice can lead to the accumulation of state mindfulness, which may subsequently foster the development of trait mindfulness over time (Goleman and Davidson, 2017; Tang, 2017; Liu et al., 2020). In other words, the deeper the mindfulness state experienced during meditation, the more likely individuals are to exhibit mindful attitudes and behaviors in everyday, non-meditative contexts (Kiken et al., 2015). Some researchers explain this by suggesting that meditation repeatedly activates neural networks associated with state mindfulness, ultimately inducing neuroplastic changes in brain function and structure that support the emergence of higher levels of trait mindfulness (Garland et al., 2010).

Conversely, other perspectives emphasize that trait mindfulness, as a personality characteristic and an inherent tendency toward mindfulness, varies naturally across individuals (Firth et al., 2023). That is, some people may exhibit a higher disposition toward trait mindfulness even in the absence of formal meditation training. In summary, state mindfulness and trait mindfulness are conceptually distinct constructs. As such, research exploring the influence of mindfulness on prosocial behavior should consider both experimental studies using mindfulness as an intervention and correlational studies treating mindfulness as a dispositional variable (Schindler and Friese, 2022). Moreover, most current studies rely on one-time behavioral measurements. However, it is unrealistic to expect that prosocial behavior observed immediately after a single mindfulness session can reliably predict behavior in specific, real-world contexts. More robust evidence requires longitudinal assessments over extended periods of time (Fleeson, 2001).

### 2.3 The present study

The intended impact of war and conflict photojournalism is to foster prosocial responses in audiences, both at the psychological level (e.g., reduced psychological distance and increased empathy; Wald et al., 2021; Liu et al., 2021) and the behavioral level (e.g., willingness to help) toward distant suffering and victims. However, research has shown that repeated exposure to images of suffering can lead to emotional desensitization among viewers (Kaplan, 2008). Meanwhile, mindfulness has been widely studied for its positive role in promoting prosociality and altruistic behavior, particularly through enhancing empathy (Winning and Boag, 2015) and increasing helping behaviors (Cameron and Fredrickson, 2015; Berry et al., 2020). Thus, mindfulness may serve as an effective intervention to enhance the prosocial impact of war photojournalism.

Helping behavior represents the core behavioral expression of prosociality, while empathy and psychological distance are key psychological mechanisms that support or inhibit such actions. This framework aligns with existing literature in social psychology and media effects research, which identifies empathy as a primary motivator for helping behavior (Batson and Shaw, 1991), and reduced psychological distance as a facilitator of empathic and prosocial responses (Trope and Liberman, 2010; Kogut and Ritov, 2005). Therefore, this study conceptualizes prosociality as comprising three dimensions: psychological distance, empathy, and helping behavior toward victims.

In sum, the primary aim of this study was to examine whether mindfulness, either as a stable trait or as a trainable state, can promote prosocial responses in the emotionally and morally challenging context of war photojournalism. Rather than testing the isolated effects of conflict imagery, the study treated exposure to such images as a constant and ecologically valid backdrop against which the impact of mindfulness could be observed. This design choice was intended to simulate real-world media encounters and to assess whether mindfulness can serve as a protective or facilitating factor when individuals engage with distressing humanitarian content.

Building on this framework, the study was conducted across three parts, each addressing a different aspect of mindfulness and its relationship to prosocial responses in the context of war photojournalism. Study 1 focuses on trait mindfulness, conceptualized as a stable personality disposition. Drawing on theoretical accounts that link trait mindfulness to increased empathy and prosocial behavior (Borghi et al., 2023), we expect that individuals with higher levels of trait mindfulness will report closer psychological proximity, greater empathy, and stronger prosocial behavioral tendencies after viewing war and conflict photojournalism.

Study 2 shifts attention to mindfulness as a trainable state, using a short-term intervention to explore the effects. Based on previous research (Hafenbrack et al., 2020; Schindler and Pfattheicher, 2023), we expect individuals who undergo the short-term mindfulness training will demonstrate decreased psychological distance, heightened empathy, and increased willingness to engage in helping behaviors among viewers of war and conflict imagery. Participants’ responses before and after the intervention are compared to assess changes in these prosocial dimensions.

Finally, study 3 examines the effects of long-term mindfulness training on the same outcomes. We expect that individuals with sustained mindfulness practice will exhibit more substantial and lasting changes in psychological distance, empathy, and prosocial behavior toward victims portrayed in war and conflict photojournalism.

## 3 Study 1: the effect of trait mindfulness on prosocial responses to war and conflict photojournalism

### 3.1 Method

#### 3.1.1 Participants

A total of 506 participants (243 males, aged 18–60 years, mean age = 34 years) were recruited for the task online^1^ in China. A power analysis using G*Power 3.1 (Faul et al., 2009) indicated that a total sample of 77 participants would be needed to detect a moderate effect size of *f^2^* = 0.15 for a linear regression analysis with three predictors, with 80% power and alpha at 0.05. Research advertisements were distributed through Wenjuanxing online platform and all participants were from the general public. No restriction was imposed on race, ethnicity, or other socially relevant characteristics. All participants were compensated financially for their time.

#### 3.1.2 Stimuli

The photojournalism materials were obtained from sources such as Xinhua News Agency, Sohu News, Phoenix News, the Associated Press, and CNN. To minimize potential biases arising from political affiliations, positions, and ethical considerations, we removed country and regional identifiers (e.g., place names and flags) from the selected photo news reports. Instead, the names of countries or regions were replaced with “X country” or “X region.” One evaluator was asked to assess whether the country/region information could still be discerned and to evaluate whether the materials exhibited political or positional biases. After evaluation, the selected 15 reports were confirmed to be suitable for the experiment. Besides, we recruited an independent sample of raters to assess each image on conflict intensity, emotional impact, and perceived realism. Each dimension was rated on a 7-point Likert scale (1 = “not at all,” 7 = “to a great extent”). Ratings confirmed that all images were perceived as depicting conflict-related content (M_conflict = 6.22, SD = 0.55) and as highly realistic (M_realism = 6.67, SD = 0.58).

#### 3.1.3 Procedure

The whole task was conducted through an online survey. Participants first viewed the 15 photo news reports and then proceeded to complete the following questionnaires. The questionnaires included measures of psychological distance, empathy, prosocial behavior, and trait mindfulness. All questionnaire items were adapted from validated scales featured in prior academic publications.

#### 3.1.4 Measures

##### 3.1.4.1 Psychological distance

The Psychological Distance Measurement Scale was derived from the article by Spence et al. (2012). This scale consists of 9 items assessing three aspects: geographic distance, social distance and uncertainty in psychological distance. Each subscale includes statements such as, “I think the area where I am might be affected by the war” (geographic distance) and “If war were to happen around me, it might have a great impact on ordinary people like me” (social distance) (see Supplementary material for the full list of items). Participants rated each item on a five-point Likert scale ranging from 1 (strongly disagree) to 5 (strongly agree). The total score and the scores of each subscale were calculated by summing up the relevant items, with higher score indicating a closer perceived psychological distance to the war (Cronbach’s *α* = 0.77).

##### 3.1.4.2 Empathy

We measured empathy using a condensed version of the Empathic Concern subscale from the Interpersonal Reactivity Index, which originally includes four dimensions: perspective taking, fantasy, empathic concern, and personal distress (Davis, 1983). Empathic concern refers to a warm emotional response, such as care, compassion, or sympathy, that arises when witnessing or becoming aware of another individual’s suffering, accompanied by a motivation to help. This subscale captures both other-oriented feelings of sympathy, concern, and compassion, as well as self-oriented feelings of sadness and fear in distressing situations. Participants rated each item on a Likert scale ranging from 1 (Strongly disagree) to 5 (Strongly agree). Empathy scores were calculated by summing the relevant items (after reverse coding where necessary), with higher scores indicating greater empathy (Cronbach’s *α* = 0.91). A detailed description of the scale is provided in Supplementary material.

##### 3.1.4.3 Prosocial behavior

In the context of reading news about war, prosocial behavior specifically involves behaviors aimed at assisting individuals suffering due to the conflict. To measure prosocial behavior in this study, we used a modified version of the prosocial behavior scale developed by Carlyle et al. (2014). The scale consists of 8 items across three dimensions: (a) support for initiatives to help war victims, (b) preferences for seeking prosocial information, and (c) intentions to engage in protective actions to assist those affected by the war. Each item was rated on a 5-point Likert scale, where 1 indicates “strongly disagree” and 5 indicates “strongly agree.” Higher scores indicate greater levels of prosocial behavior (Cronbach’s α = 0.92). For a detailed description of the scale, please see Supplementary material.

##### 3.1.4.4 Trait mindfulness

To assess participants’ trait mindfulness levels, we used the Five Facet Mindfulness Questionnaire (FFMQ; Deng et al., 2011), a 39-item scale that evaluates five key facets of mindfulness: observing, describing, non-judging of inner experience, non-reactivity to inner experience, and acting with awareness (please see Supplementary material). Each subscale includes statements such as, “I notice the smells and aromas of things” for observing, and “I tell myself I should not be feeling the way I’m feeling” for non-judging. Participants rated each item on a five-point Likert scale ranging from 1 (never or very rarely true) to 5 (very often or always true). The total score, as well as scores for each subscale, were calculated by summing individual item responses. Higher scores on the FFWQ reflect a stronger tendency to maintain present-moment attention and awareness in everyday life (Cronbach’s α = 0.98). The FFMQ has demonstrated good psychometric properties in prior studies with strong construct validity and internal consistency.

### 3.2 Results

To examine whether trait mindfulness predicted psychological distance, empathy, and prosocial behavior, three separate linear regression analyses were conducted (Table 1).

**Table 1:** Regression coefficients for trait mindfulness scores predicting psychological distance, empathy, and prosocial behavior in Study 1.

Variable	*β*	*t*(504)	*F*(1,504)	*R* ^2^	*p*
Psychological distance	0.415	10.245	104.956	0.172	< 0.001
Empathy	0.382	9.291	86.324	0.146	< 0.001
Prosocial behavior	0.413	10.182	103.676	0.171	< 0.001

For psychological distance, the regression model was significant, *F*(1, 504) = 104.956, *p* < 0.001, indicating that the trait mindfulness accounted for a significant portion of the variance in psychological distance. Although it explained 17.2% of the variance (*R*^2^ = 0.172), the relationship was statistically significant with a moderate effect size, as trait mindfulness positively predicted psychological distance, *β* = 0.415, *t*(504) = 10.245, *p* < 0.001.

For empathy, the regression model was also significant, *F*(1, 504) = 86.324, *p* < 0.001, with the trait mindfulness explaining 14.6% of the variance (*R*^2^ = 0.146). Trait mindfulness significantly predicted empathy scores, *β* = 0.382, *t*(504) = 9.291, *p* < 0.001.

Similarly, the regression model was significant for prosocial behavior (*F*(1, 504) = 103.676, *p* < 0.001, *R*^2^ = 0.171), where trait mindfulness was a positive predictor of prosocial behavior (*β* = 0.413, *t*(504) = 10.182, *p* < 0.001.).

### 3.3 Discussion

These findings suggest that individuals with higher levels of trait mindfulness experienced shorter psychological distance, exhibited greater empathy, and engaged in more frequent prosocial behaviors. This aligns with prior research showing that trait mindfulness facilitates emotional regulation (Brown and Ryan, 2003; Liu et al., 2022; Chen et al., 2023) and perspective-taking (Feldman et al., 2007), both of which are foundational to prosocial engagement. Thus, trait mindfulness emerges as an important disposition influencing the prosocial responses of audiences exposed to war and conflict photojournalism. Given that trait mindfulness can be developed through repeated state mindfulness practice (Kiken et al., 2015), and that state mindfulness has been empirically linked to increased prosocial tendencies (Donald et al., 2019), it is reasonable to hypothesize that even short-term mindfulness training may enhance prosocial responses to media depicting human suffering. Therefore, Study 2 aims to investigate whether a brief mindfulness-based intervention can foster greater prosociality in audiences exposed to war and conflict news.

## 4 Study 2: the effect of short-term mindfulness training on prosocial responses to war and conflict photojournalism

### 4.1 Method

#### 4.1.1 Participants

Seventy-two college students (4 males, aged 18–25 years, mean age = 20 years) from Jinan University were recruited for this experiment. *A priori* power analysis was conducted using G*Power 3.1 to determine the required sample size for a paired-samples t-test. Assuming a medium effect size (Cohen’s 𝑑 = 0.50), an alpha level of 0.05, and desired power of 0.80, the analysis indicated that a total of 34 participants would be required to detect a statistically significant difference between the two conditions. All participants reported no prior experience with emotional training, positive thinking, or meditation. All had normal or corrected-to-normal vision. Consent forms were obtained from all participants before they took part in the experiment, and the participants were compensated financially for their time.

Ten participants did not complete the training session and were therefore excluded from the final analysis. The resulting sample for this study consisted of 62 participants (4 males, aged 18–25 years, mean age = 20 years).

#### 4.1.2 Stimuli

In this experiment, two sets of photo news reports were used because participants completed two phases: a pre-test phase before mindfulness training and a post-test phase after training. To avoid exposing participants to the same materials across phases, we selected another 15 photo news reports to form a new stimulus set. These images were selected and subjected to the same evaluation procedure as before, and were confirmed to depict high levels of conflict (M_conflict = 6.22, SD = 0.55) and realism (M_realism = 6.51, SD = 0.42) using the same 7-point Likert scales. One set was presented during the pre-test phase and the other during the post-test phase. To control for potential order effects, the assignment of the two sets (Set A and Set B) to the pre- or post-test phase was counterbalanced across participant. That is, half of the participants viewed Set A during the pre-test and Set B during the post-test, while the other half viewed them in the reverse order. This design ensured that any observed effects could not be attributed to the specific content of one stimulus set or to stimulus order.

The mindfulness training material consisted of a one-hour audio recording delivered via an audio-based app. The content was adapted from Mindfulness: A Practical Guide to Finding Peace in a Frantic World by Williams and Penman (2012), which is widely used in mindfulness-based cognitive therapy (MBCT). This guided audio session was designed for individuals without prior meditation experience and included core mindfulness practices such as breathing exercises, body scanning, and focused awareness.

#### 4.1.3 Procedure

Before the short-term meditation training, participants completed a pre-test similar to that in Study 1. The pre-test included viewing a set of selected photo news reports (set A or set B) and completing a series of questionnaires measuring psychological distance, empathy, and prosocial behavior. Following the pre-test, participants underwent a one-week mindfulness training program. Each day, they engaged in a one-hour mindfulness meditation session conducted in a psycho-group counseling room. During the process, participants were asked to follow the instruction to focus on a selected object, such as the body or the breath, monitoring the activity of the mind, and developing a non-judgmental awareness of experience. The training did not include any reference to loving-kindness, compassion, empathy, prosocial behavior, or related terms.

After completing the seven-day meditation training course, participants completed a post-test using the same questionnaires as the pre-test, but with photo news reports from the alternate set.

### 4.2 Results

Paired *t*-tests were used to compare pre- and post-test scores on psychological distance, empathy, and prosocial behavior (Table 2).

**Table 2:** Paired samples *t*-tests comparing pre- and post-test measures in Study 2.

Variable	Mean difference	SE difference	*t*(61)	*p*	Cohen’s *d*
Psychological distance	−1.483	0.371	−4.004	< 0.001	−0.474
Empathy	−0.677	0.448	−1.514	0.135	−0.143
Prosocial behavior	−0.451	0.453	−0.996	0.323	−0.101

Results showed a significant reduction in psychological distance, with participants reporting a lower score on the pre-test (*M* = 27.27, SE = 0.38) than the post-test (*M* = 28.76, SE = 0.42), *t*(61) = −4.004, *p* < 0.001, Cohen’s *d* = −0.474. However, there were no significant changes in empathy (*t*(61) = −1.514, *p* = 0.135, Cohen’s *d* = −0.143) or prosocial behavior (*t*(61) = −0.996, *p* = 0.323, Cohen’s *d* = −0.101).

### 4.3 Discussion

These results suggest that short-term mindfulness meditation was effective in reducing psychological distance, but it did not significantly affect empathy or prosocial behavior. This indicates that the effect of state mindfulness induced by short-term training on the prosociality of audiences exposed to war and conflict photojournalism is limited, primarily manifesting in reduced psychological distance without significantly enhancing empathy or intentions for altruistic action. One possible explanation for these findings could be the insufficient duration of the mindfulness training. Prior research has shown that more sustained mindfulness practice is often required to produce measurable changes in emotional empathy and altruistic behavior (Luberto et al., 2018). While brief interventions may increase present-focused awareness and lessen emotional distress, they often fall short in nurturing the deeper sense of compassion required for meaningful prosocial engagement. Therefore, Study 3 will further investigate whether long-term meditation training can yield greater improvements in prosocial outcomes.

## 5 Study 3: the effect of long-term mindfulness training on prosocial responses to war and conflict photojournalism

### 5.1 Method

#### 5.1.1 Participants

Another seventy-two participants (33 males, aged 18–50 years, mean age = 26 years) with long-term meditation experience were recruited through a mindfulness meditation training group on Douban and Xiaohongshu, two Chinese social media platforms. A power analysis using G*Power 3.1 indicated that a total sample of 128 participants (64 for each group) would be needed to detect a moderate effect size of Cohen’s 𝑑 = 0.50 for an independent-samples t-test, with 80% power and alpha at 0.05. All participants had been engaged in mindfulness meditation practice for at least 6 months, with no history of severe psychological disorders or psychiatric diagnoses. All participants were compensated financially for their time.

#### 5.1.2 Procedure

Participants followed a procedure similar to that in Study 1, first browsing 15 photo news reports and then completing assessments of psychological distance, empathy, and prosocial behavior. They were also asked to report the duration of their prior meditation experience, recorded in years. For consistency, the duration was rounded to the nearest full year, i.e., less than 6 months was counted as 0 years, while 6 months or more was rounded up to 1 year.

### 5.2 Results

To examine the effect of long-term mindfulness experience, independent samples t-tests were conducted to compare the long-term meditator group (participants with long-term meditation experience) and the non-meditator group (participants with no experience from Study 2) across three aspects: psychological distance, empathy, and prosocial behavior (Table 3).

**Table 3:** Independent samples t-tests comparing long-term meditation group and naïve group.

Variable	Mean difference	SE difference	*t*(142)	*p*	Cohen’s *d*
Psychological distance	0.319	0.446	0.716	0.475	0.120
Empathy	−0.111	0.866	−0.128	0.898	−0.010
Prosocial behavior	−1.528	0.635	−2.406	0.017	−0.399

The analysis revealed that there was a significant difference in prosocial behavior between long-term meditators and non-meditators, *t*(142) = −2.406, *p* = 0.017, Cohen’s *d* = −0.399. Long-term meditators exhibited significantly more prosocial behavior (*M* = 34.58, SE = 0.42) compared to non-meditators (*M* = 33.06, SE = 0.48). However, no significant differences were observed for psychological distance (*t*(142) = 0.716, *p* = 0.475, Cohen’s *d* = 0.120) or empathy (*t*(142) = −0.128, *p* = 0.898, Cohen’s *d* = −0.010).

Additionally, a positive correlation was found between the length of meditation practice and prosocial behavior (*r*(72) = 0.35, *p* = 0.001) for long-term meditators, indicating that longer meditation practice was associated with more frequent prosocial behaviors. In contrast, no significant correlations were observed between the length of meditation practice and psychological distance, *r*(72) = −0.12, *p* = 0.17, or empathy, *r*(72) = 0.08, *p* = 0.27.

### 5.3 Discussion

According to the above findings, long-term mindfulness training played a significant role in promoting prosocial behavior, even though it did not have a significant impact on psychological distance or empathy. This suggest that extended practice may foster prosociality through mechanisms beyond emotional resonance-such as enhanced self-regulation, value alignment, or moral awareness (Dahl et al., 2015). The positive association between training duration and willingness to help further supports this idea. In emotionally taxing media environments like war and conflict journalism, where audiences often disengage or experience compassion fatigue (Kaplan, 2008), sustained mindfulness may help cultivate a more resilient and ethically grounded response. These results highlight the potential of long-term mindfulness as a tool for supporting prosocial engagement in contexts where empathy alone may not be enough.

## 6 General discussion

War and conflict photojournalism remains a powerful tool for revealing human suffering and prompting moral reflection. Its impact depends on the viewer’s ability to emotionally engage with people who are geographically and psychologically distant. However, repeated exposure to such content can lead to compassion fatigue, weakening the connection between emotional reactions and prosocial behavior (Kaplan, 2008). To better understand what supports or hinders this process, we examined how trait mindfulness, short-term mindfulness training, and long-term mindfulness practice influence audience responses to war-related imagery.

We found that individuals with higher trait mindfulness reported lower psychological distance, stronger empathy, and greater willingness to help. These findings are in line with previous research showing that mindfulness supports perspective-taking and emotional sensitivity (Brown and Ryan, 2003; Condon et al., 2013). Importantly, our results go further by showing that trait mindfulness can promote not only empathic feelings but also more effortful, costly prosocial behaviors, such as volunteering or donating, when viewers are exposed to morally urgent situations like war photography (Schindler and Pfattheicher, 2023). This suggests that the prosocial impact of mindfulness may be amplified by emotionally powerful stimuli.

Short-term mindfulness training, on the other hand, had more limited effects. It helped reduce psychological distance but did not significantly increase empathy or lead to prosocial action. This supports earlier findings that brief interventions can improve present-moment awareness and emotional regulation, but may not be enough to trigger deeper behavioral change (Kiken et al., 2015). The nature of the prosocial behavior matters as well. As noted by Berry et al. (2020), lower-cost behaviors, like offering a seat, are more responsive to short-term mindset shifts, while high-cost actions tend to require sustained motivation. Our results align with this view: short-term mindfulness may prepare individuals cognitively, but not yet move them to act.

Interestingly, participants with long-term mindfulness practice were more likely to engage in prosocial behavior, even though they did not report greater empathy or psychological closeness to victims. This points to a different pathway. Rather than relying solely on emotional resonance, long-term mindfulness may foster prosociality by enhancing emotional regulation and reducing avoidance. As suggested by Schindler and Friese (2022), mindfulness can help individuals tolerate discomfort, allowing them to stay present with distressing content without shutting down emotionally. This capacity to remain open and engaged may explain why long-term practitioners were more willing to help, even in emotionally challenging contexts.

Taken together, these findings suggest that mindfulness, especially when developed over time, can support meaningful social engagement with others’ suffering. While brief training may increase awareness and reduce defensiveness, long-term practice appears to provide the stability and resilience needed to act compassionately in difficult circumstances. This highlights the importance of not only what mindfulness is, but how it is cultivated—offering insights for both theory and practice.

### 6.1 Theoretical contributions

The present research makes theoretical contributions in several ways. To begin with, it extends the application of mindfulness within the domain of media effects research. By introducing both trait and state mindfulness into the study of audience responses to war photojournalism, we reveal how mindfulness, as a psychological self-regulation mechanism, influences psychological distance, empathy, and prosocial behavior in the face of violent imagery. Particularly, the finding that trait mindfulness interacts with situational stimuli (i.e., war photography) addresses a gap in existing literature.

Furthermore, the study deepens the motivational mechanism explanation of prosocial behavior. By distinguishing the differential effects of short-term and long-term mindfulness training, our findings support the “multiple pathways hypothesis” of prosocial motivation. Specifically, short-term mindfulness appears to facilitate low-cost prosocial behaviors by regulating emotional avoidance, whereas long-term mindfulness fosters high-cost behaviors, such as donation and volunteering, by enhancing executive self-control. This insight sheds light on the non-linear relationship between mindfulness and prosocial behavior and offers a more nuanced understanding of how mindfulness practices may selectively activate different motivational pathways.

Moreover, this research challenges the traditional empathy-driven model of prosociality. Although long-term mindfulness training increased costly helping behaviors, it did not significantly enhance empathy or reduce psychological distance. This finding questions the classic assumption that empathy necessarily precedes helping and suggests that mindfulness may promote behavioral change through non-empathic pathways, such as emotional regulation and self-control. It provides new empirical evidence for the debate between altruistic and egoistic motivations underlying prosocial behavior, enriching theoretical discussions on the complexity of helping behavior mechanisms.

Finally, by linking mindfulness with media-induced prosociality, this research contributes to a more interdisciplinary understanding of how individual psychological traits interact with media content to shape ethical responses. This perspective invites future work at the intersection of psychology, communication studies, and social ethics-particularly in emotionally intense domains such as humanitarian journalism, public crisis narratives, and digital media environments.

### 6.2 Practical contributions

First, the finding that audience psychological traits moderate responses to war imagery informs media ethics and strategy. Media outlets could integrate mindfulness prompts, such as brief guided reflections, into conflict reporting to ease compassion fatigue and reduce reliance on emotional sensationalism. For instance, attaching short mindfulness exercises to news stories may help audiences manage distress while sustaining attention to humanitarian issues.

Second, the results suggest differentiated advocacy strategies based on audience mindfulness levels. For individuals with high trait mindfulness, directly presenting the harsh realities of war victims may effectively trigger donations. For the general public, integrating long-term mindfulness training (e.g., sustained meditation programs) may better foster willingness to engage in high-cost prosocial actions like volunteering and substantial giving.

Third, our findings point to opportunities for designing more tailored public engagement programs. For example, short-term mindfulness interventions may be suitable for prompting low-barrier behaviors, such as sharing information or signing petitions, especially in fast-paced digital contexts. In contrast, long-term mindfulness programs may be more effective for cultivating deeper engagement, including volunteer participation, charitable giving, or long-term advocacy.

Fourth, the results have implications for training and policy development. Mindfulness-based modules could be integrated into journalism ethics education, helping reporters build emotional resilience and maintain compassionate attention when covering traumatic events. Similarly, humanitarian organizations and nonprofits may consider incorporating mindfulness into staff development or public outreach, supporting both internal well-being and external engagement.

Finally, the findings contribute to trauma-informed communication practices. As crises like war, displacement, and environmental disaster increasingly dominate media narratives, equipping audiences and communicators with emotional regulation tools such as mindfulness may help sustain engagement without emotional overload. This opens avenues for developing emotionally ethical media designs that support both empathy and resilience.

### 6.3 Limitations

There are several limitations in our study. First, the absence of a control group in Study 2 limits our ability to draw firm causal conclusions about the effects of mindfulness. Without a no-intervention comparison condition, we cannot fully rule out time-related factors or other uncontrolled influences. Nonetheless, to reduce potential confounds, we used two non-overlapping sets of war images in the pre- and post-tests and counterbalanced their order. Moreover, the mindfulness intervention was framed in a neutral way (e.g., breath awareness, present-moment focus), with no mention of empathy or prosociality, which may have reduced demand characteristics. Future research should adopt randomized controlled trials to improve causal inference.

Second, mindfulness was examined solely in the context of conflict-related imagery, without comparison to neutral or alternative negative images. This limits our ability to disentangle the effects of mindfulness from the emotional salience of the content. However, this was an intentional design choice, as our goal was to examine whether mindfulness can promote prosocial responses within emotionally and morally charged media environments. Future studies could use factorial designs to systematically vary both image type and intervention, offering more precise insight into how media content and mindfulness interact to influence audience responses.

Third, demographic differences across the three studies may have introduced some variability. This was an intentional choice reflecting each study’s specific aims: Study 1 recruited a diverse community sample to examine trait mindfulness broadly; Study 2 focused on a more homogeneous group of university students to better control the short-term intervention; Study 3 involved experienced long-term meditators, mostly from self-selected mindfulness communities. While these differences may limit generalizability and direct comparison between studies, supplementary analyses in Study 3 showed no significant age-related differences in prosocial behavior, suggesting limited age effects. Additionally, Study 2 had an imbalanced gender distribution, with only four male participants, which restricted our ability to analyze gender effects. Given the small number of males and the within-subject design focused on pre-post changes, any gender-related influence likely added random variation rather than systematic bias. Future research should aim for more balanced gender and age representation to explore these factors more thoroughly.

Fourth, in Studies 2 and 3, we did not measure participants’ trait mindfulness, so we cannot determine whether short-term or long-term mindfulness training can promote the development of trait mindfulness. Although this was not the primary focus of our research, future studies could investigate how the duration of training influences the transformation between different forms of mindfulness. Another limitation is that we relied on self-reports to measure prosocial behavior (e.g., “Would you be willing to donate?”), which may be subject to social desirability bias. Future research could include behavioral experiments with real donation options or track participation in volunteer activities over time.

Lastly, while long-term mindfulness training increased prosocial behavior, it did not enhance empathy. The psychological mechanisms underlying this effect remain unclear: whether it is due to emotional desensitization, improved executive control, or habitual altruism. The lack of physiological indicators (e.g., heart rate variability, neuroimaging data) or cognitive tasks (e.g., emotional regulation tests) makes it difficult to differentiate whether the observed effects result from “self-regulation” or “empathy attenuation.” This presents an important avenue for future research.

## Data Availability

The raw data supporting the conclusions of this article will be made available by the authors, without undue reservation.

## Footnotes

^1^https://www.wjx.cn/
