# Supplementary material for: Mindfulness and media-driven prosociality: effects of trait and state mindfulness on responses to conflict photojournalism
Source: Front Psychol. 2025 Aug 13;16:1619688. doi: 10.3389/fpsyg.2025.1619688 (PMC12382450; doi:10.3389/fpsyg.2025.1619688)
Supplement: Supplementary file 1 [file Data_Sheet_1.docx]

Supplementary Material

All questionnaires used in this study are listed below. Some items were directly adapted from the original scales, while others were modified to better align with the war-related context of the study.

## Psychological Distance Measurement Scale (Items 1-8)

1. My local area is likely to be affected by the war.

2. Wars will mostly affect areas that are far away from where I live. (reverse coded)

3. Wars will mostly affect developing countries other than my nation.

4. If a war were to break out nearby, it would have a big impact on people like me.

5. I think war is unjust.

6. I am uncertain that war is really happening. (reverse coded)

7. The seriousness of the war is often exaggerated. (reverse coded)

8. It is uncertain what the effects of the war will be. (reverse coded)

## Empathic Concern Items (Items 1-9)

1. I often have tender, concerned feelings for people less fortunate than me.

2. Sometimes I don't feel very sorry when I see images in the above photo news. (reverse coded)

3. When others are in trouble, I do not usually feel worried for them. (reverse coded)

4. I feel scared after seeing the above photo news.

5. When I see someone being taken advantage of, l feel kind of protective towards them.

6. Other people's misfortunes do not usually disturb me a great deal. (reverse coded)

7. After seeing the photo news, I feel sad.

8. When I see someone being treated unfairly, I sometimes don't feel very much pity for them. (reverse coded)

9. I would describe myself as a pretty soft-hearted person.

## Prosocial Behavior Items (Items 1-8)

1. I would like more information about how to help war victims.

2. I would like to better understand how to provide useful resources and support for war victims.

3. I would like to learn more about how wars affect the psychological and emotional well-being of victims.

4. I hope that international organizations can offer telephone or online help hotlines for war victims.

5. I hope that international organizations can provide shelters for war victims.

6. I hope that international organizations can offer free counseling services to war victims.

7. If I have the chance, I am willing to donate money to international organizations that protect war victims.

8. If I have the chance, I am willing to participate in public service activities related to promoting world peace.

## Five Facet Mindfulness Questionnaire (Items 1-39)

1. When I’m walking, I deliberately notice the sensations of my body moving.

2. I’m good at finding words to describe my feelings.

3. I criticize myself for having irrational or inappropriate emotions.

4. I perceive my feelings and emotions without having to react to them.

5. When I do things, my mind wanders off and I’m easily distracted.

6. When I take a shower or bath, I stay alert to the sensations of water on my body.

7. I can easily put my beliefs, opinions, and expectations into words.

8. I don’t pay attention to what I’m doing because I’m daydreaming, worrying, or otherwise distracted.

9. I watch my feelings without getting lost in them.

10. I tell myself I shouldn’t be feeling the way I’m feeling.

11. I notice how foods and drinks affect my thoughts, bodily sensations, and emotions.

12. It’s hard for me to find the words to describe what I’m thinking.

13. I am easily distracted.

14. I believe some of my thoughts are abnormal or bad and I shouldn’t think that way.

15. I pay attention to sensations, such as the wind in my hair or sun on my face.

16. I have trouble thinking of the right words to express how I feel about things.

17. I make judgments about whether my thoughts are good or bad.

18. I find it difficult to stay focused on what’s happening in the present.

19. When I have distressing thoughts or images, I “step back” and am aware of the thought or image without getting taken over by it.

20. I pay attention to sounds, such as clocks ticking, birds chirping, or cars passing.

21. In difficult situations, I can pause without immediately reacting.

22. When I have a sensation in my body, it’s difficult for me to describe it because I can’t find the right words.

23. It seems I am “running on automatic” without much awareness of what I’m doing.

24. When I have distressing thoughts or images, I feel calm soon after.

25. I tell myself that I shouldn’t be thinking the way I’m thinking.

26. I notice the smells and aromas of things.

27. Even when I’m feeling terribly upset, I can find a way to put it into words.

28. I rush through activities without being really attentive to them.

29. When I have distressing thoughts or images, I am able just to notice them without reacting.

30. I think some of my emotions are bad or inappropriate and I shouldn’t feel them.

31. I notice visual elements in art or nature, such as colors, shapes, textures, or patterns of light and shadow.

32. My natural tendency is to put my experiences into words.

33. When I have distressing thoughts or images, I just notice them and let them go.

34. I do jobs or tasks automatically without being aware of what I’m doing.

35. When I have distressing thoughts or images, I judge myself as good or bad, depending what the thought/image is about.

36. I pay attention to how my emotions affect my thoughts and behavior.

37. I can usually describe how I feel at the moment in considerable detail.

38. I find myself doing things without paying attention.

39. I disapprove of myself when I have irrational ideas.
